# Wound complications after primary and repeated midline, transverse and modified Makuuchi incision: A single-center experience in 696 patients

**DOI:** 10.1097/MD.0000000000025989

**Published:** 2021-05-21

**Authors:** Sebastian Hempel, Anne Kalauch, Florian Oehme, Steffen Wolk, Thilo Welsch, Jürgen Weitz, Marius Distler

**Affiliations:** Department of Visceral, Thoracic and Vascular Surgery, University Hospital and Faculty of Medicine Carl Gustav Carus, Technische Universität Dresden, Dresden, Germany.

**Keywords:** fascial dehiscence, incisional hernia, re-incision, relaparotomy, wound complication

## Abstract

There are 3 main types of incisions in major open, elective abdominal surgery: the midline incision (MI), the transverse incision (TI) and the modified Makuuchi incision (MMI). This study aimed to compare these approaches regarding wound complications and hernias, with a special focus on suture material and previous laparotomies.

Patients who underwent elective abdominal surgery between 2015 and 2016 were retrospectively analyzed. Uni- and multivariate analyses were computed using stepwise binary and multifactorial regression models.

In total, 696 patients (406 MI, 137 TI and 153 MMI) were included. No relevant differences were observed for patient characteristics (e.g., sex, age, body mass index [BMI], American Society of Anesthesiologists [ASA] score). Fewer wound complications (TI 22.6% vs MI 33.5% vs MMI 32.7%, *P* = .04) occurred in the TI group. However, regarding the endpoints surgical site infection (SSI), fascial dehiscence and incisional hernia, no risk factor after MI, TI, and MMI could be detected in statistical analysis. There was no difference regarding the occurrence of fascial dehiscence (*P* = .58) or incisional hernia (*P* = .97) between MI, TI, and MMI. In cases of relaparotomies, the incidence of fascial dehiscence (*P* = .2) or incisional hernia (*P* = .58) did not significantly differ between the MI, TI, or MMI as well as between primary and reincision of each type. On the other hand, the time to first appearance of a hernia after MMI is significantly shorter (*P* = .03) than after MI or TI, even after previous laparotomy (*P* = .003).

In comparing the 3 most common types of abdominal incisions and ignoring the type of operative procedure performed, TI seems to be the least complicated approach. However, because the incidence of fascial dehiscence and incisional hernia is not relevantly increased, the stability of the abdominal wall is apparently not affected by relaparotomy, even by repeated MIs, TIs, and MMIs. Therefore, the type of laparotomy, especially a relaparotomy, can be chosen based on the surgeon's preference and planned procedure without worrying about increased wound complications.

## 1 Introduction

Although the minimally invasive technique has gained importance in abdominal surgery in recent years, conventional access is still standard procedure for many indications. Midline (MI) and transverse incisions (TI) are the methods most widely used because they provide optimal access in most cases and can be easily extended if necessary. Modified Makuuchi incision (MMI) is a common incision in right-sided hepatectomy or liver transplantation. The choice of incision is primarily based on surgeon preference. However, MI is the most commonly used approach in visceral surgery. After MI, around 10% to 15% of patients develop postoperative incisional hernias and about 15% to 30% postoperative wound infections.^[1]^ In randomized controlled trials with sufficient follow-up time, these complication rates are even higher.^[2–5]^

There is ongoing debate as to which suturing techniques and suture materials are best for achieving definitive abdominal wound closure while minimizing the risk of short- and long-term complications. The STITCH trial recommends a small bite technique with polydioxanone suture [PDS] Plus 2-0 suture for prevention of incisional hernia without increasing adverse events.^[6]^ According to the results of the POVATI-trial, MI and TI can be used equivalently regarding postoperative morbidity.^[7]^ Comparative data for MI, TI, and MMI regarding wound complications is limited. So far, the clinical outcome of these 3 approaches has only been compared in the context of donor hepatectomy in living donor liver transplantation.^[8]^

The aim of this study was to compare MI, TI, and MMI with regard to wound complications and frequency of incisional hernia, with a special focus on suture material, previous laparotomies and relaparotomies using the same incision.

## 2 Methods

### 2.1 Study design and patients

The study was designed as a retrospective observational study. Included were all patients who underwent elective abdominal surgery with MI, TI, and MMI for various indications between January 2015 and December 2016 at the Department of Visceral, Thoracic and Vascular Surgery, University Hospital Carl Gustav Carus, Technische Universität Dresden, Germany. Cases of emergency laparotomy were not included.

The medical records for each case were analyzed retrospectively using our prospective database. In addition, postoperative events, clinical outcomes and ambulatory course were recorded prospectively and analyzed retrospectively.

This retrospective monocentric study was conducted in accordance with the Declaration of Helsinki and informed consent was obtained from the all included patients. The experimental protocol of the study was approved by the local ethics committee at the Carl Gustav Carus University Hospital (decision number EK188052017).

### 2.2 Definitions

Relaparotomies or abdominal re-incisions were defined as all cases in which patients had already undergone a laparotomy in the past, regardless of the site of incision.

The term “wound complications” indicates any problems in wound healing without further precise definition. Considered for further analysis were only the following clinical relevant wound complications:

Surgical site infections (SSI) were defined as local epifascial wound infections within the hospital stay caused by seroma or hematoma necessitating of any wound intervention (e.g., wound irrigation or reoperation).

Fascial dehiscence was defined as a complete disruption of the fascial closing or a significantly gap between the margins of the fascia within the first postoperative days with the need of reoperation.

Incisional hernias were determined as a hernia documentation in the medical record by regular follow-up consultations or a reoperation due the hernia. The “time to hernia” was defined as the time between the index operation and the last follow-up contact with first appearance of incisional hernia.

### 2.3 Operative technique

All patients were given a preincisional antibiotic prophylaxis with 1.5 g cefuroxime and 0.5 g metronidazole 30 minutes before incision. After skin preparation using a mix of the antiseptic chlorhexidine and alcohol, the skin was incised either with a conventional or monopolar scalpel and the abdominal wall was dissected by electrocoagulation. The TI ran 2 fingers beneath the costal border in a concave alignment to the umbilicus. For the MI, the skin was incised in a semicircular direction at the level of the umbilicus. For the MMI, the skin was incised in the midline and at the level of umbilicus in a transverse direction to the right side. MIs were closed with a single-layer technique and TIs and MMIs with a two-layer technique using a continuous monofilament absorbable PDS Plus 2-0 suture (PDS Plus 2-0, 70 cm, MH-1 (31 mm); Ethicon, Cincinnati, OH, USA). In cases of relaparotomy, the multiple incisions were closed in the same way but using the PDS 1 Plus suture (PDS 1 Plus, 90 cm, CT 40 mm), Ethicon, Cincinnati, OH, USA).

### 2.4 Statistical analysis

For statistical calculation and obtaining data plots, IBM SPSS 25 (SPSS Statistics V25, IBM Corporation, Armonk, New York) was used. The significance level for all calculations was set at *P* = .05. Fisher's exact test and the unpaired *t*-test were used to test categorical or quantitative variables. The quantitative variables age, body mass index (BMI), length of hospital stay, intensive care unit stay and follow-up time were expressed as median with interquartile range (IQR). Follow-up time was defined from the date of surgery to last patient contact.

Uni- and multivariate analyses were computed using stepwise binary and multifactorial regression models. The following variables were considered for univariate analysis: age, sex, American Society of Anesthesiologists [ASA] score and comorbidities (e.g., diabetes, nicotine and alcohol abuse, previous laparotomies), BMI ≥25 kg/m^2^, surgical procedure, suture and intraoperative drainage. All significantly different variables in univariate analysis and all those considered clinically relevant were included in the multivariate analysis. The multiple linear regression model considered the following variables for all cases and each incision separately: patient-related risk factors (BMI ≥25 kg/m^2^, diabetes, nicotine abuse, alcohol abuse, previous laparotomy or previous laparotomy via the same incision) and operation-related risk factors (e.g., suture and intraoperatively inserted drain). The curves of the probability of incisional hernia were determined using the Kaplan–Meier method. Differences between the curves were identified using the log-rank test.

## 3 Results

### 3.1 Patient cohort

In total, 696 patients underwent elective abdominal surgery for different indications within the study period. The median age of our patient cohort was 66 years (IQR 56–74 years). The majority of patients were male (n = 433, 62.2%), and the median body mass index (BMI) was 25.05 kg/m^2^ (IQR 22.6–29 kg/m^2^). The majority of our patient cohort were patients with a mild systemic disease (ASA 2, n = 226, 35.5%) and those with a severe systemic disease that was not life-threatening (ASA 3, n = 379, 59.6%).

The most frequent concomitant diagnosis and symptoms at the time of presentation were diabetes (n = 173, 24.9%), alcohol abuse (n = 137, 21.5%), and nicotine abuse (n = 140, 20.3%). More than 50% of all operated patients (n = 366, 52.6%) had already been laparotomized in the past; nearly a quarter of them (n = 168, 24.1%) had even undergone a laparotomy via the same incision (Table 1).

**Table 1:** Patient characteristics.

Parameters	MIn = 406	TIn = 137	MMIn = 153	*P* value
Sex [n (%)]				.02
m	270 (66.5)	76 (55.5)	87 (56.9)	
w	136 (33.5)	61 (44.5)	66 (43.1)	
Median age [yrs] (IQR)	66 (56–75)	67 (57–74)	65 (55–73)	.46
Median BMI [kg/m^2^] (IQR)	25 (22.5–28.6)	24.6 (22.2–27.8)	26.2 (23.3–29)	.69
ASA Score [n (%)]				.13
I	9 (2.2)	5 (3.7)	3 (2)	
II	120 (29.6)	49 (35.8)	57 (37.2)	
III	218 (53.7)	74 (54)	87 (56.9)	
IV	13 (3.2)	1 (0.7)	0	
NA	46 (11.3)	8 (5.8)	6 (3.9)	
Comorbidities [n (%)]^∗^				
Diabetes	94 (23.2)	45 (32.8)	34 (22.2)	.06
Nicotine abuse	87 (21.8)	32 (23.4)	21 (13.7)	.07
Alcohol abuse	85 (23.7)	23 (16.8)	29 (19)	.29
Previous laparotomy	205 (50.5)	61 (44.5)	100 (65.4)	<.01
Previous laparotomy via same incision	108 (26.6)	22 (16.1)	38 (24.8)	.04
Type of surgery				<.001
Liver resection	3 (0.7)	0	150 (98)	
Pancreatic surgery	27 (6.7)	130 (95)	1 (0.7)	
Gastrectomy	95 (23.4)	2 (1.4)	0	
Colorectal resection	232 (57.1)	1 (0.7)	1 (0.7)	
Others	49 (12.1)	4 (2.9)	1 (0.7)	
Median length of hospital stay [d] (IQR)	20 (14–36)	19 (13–28)	17 (11–27)	.06
ICU stay[d] (IQR)	3 (2–7)	3 (2–6)	3 (2–6)	.68
Median follow-up [months] (IQR)	19.7 (6–41)	14.2 (8.7–28.4)	16.2 (6.1–36.7)	.22

In 406 cases, we performed an MI, mostly for colorectal surgery (n = 232, 57.1%) and gastrectomies (n = 95, 23.4%). In this group, the median length of hospital stay was 20 (14–36) days (IQR) and the postoperative median follow-up time was 600 (182–1256) days (IQR).

TI (n = 137) was primarily performed for pancreatic surgery (n = 130, 95%). After TI, patients were in in-patient treatment for a median of 19 (13–28) days (IQR). The median follow-up was 432 (263–864) days (IQR).

An MMI was performed in 153 cases. In almost 100% of the cases (n = 150, 98%), we performed this incision for major liver resections. The median length of hospital stay was 17 (11–27) days (IQR). In this group, the median follow-up was 493 (186–1116) days (IQR).

### 3.2 Operation characteristics and wound complications

In all cases, a preoperative antibiotic prophylaxis with 1.5 g cefuroxime and 0.5 g metronidazole was given. In general, abdominal wall closure after primary laparotomy and relaparotomy was achieved with a PDS Plus 2-0 and PDS 1 Plus suture, respectively. In 272 (39.1%) cases, we used a PDS Plus 2-0 suture and in 420 (60.3%) a PDS 1 Plus suture. In 4 patients, (0.6%), both sutures were used in combination. The discrepancy between the cases of relaparotomy (n = 366) and the actual use of PDS 1 Plus suture (n = 420) can be explained by the individual decision of the surgeon in difficult fascial conditions.

For TI (n = 79, 57.7%), we used a PDS Plus 2-0 suture more than for MI (n = 142, 35%) or MMI (n = 51, 33.3%) (Table 2). An intra-abdominal drain was inserted in almost every operation (n = 676, 97.1%). Overall, wound complications in general occurred in 217 cases (31.2%). The rate of wound complications in the TI group (n = 31, 22.6%) was significantly (*P* = .04) lower than in the MI (n = 136, 33.5%) or MMI group (n = 50, 32.7%). Especially, there occurred significantly less SSI (n = 23, *P* = .04) in the TI group. Therefore, fewer wound interventions were required in the TI group (n = 23, 16.8%, *P* = .08). There were only little differences between the groups in the need and the number of reoperations due wound complications. Overall, the incidence of fascial dehiscence or incisional hernias was less than 10% of all patient cases during the follow-up period. The lowest rate of fascial dehiscence was observed in the TI group (n = 5, 3.6%, *P* = .58). The incidence of incisional hernia was nearly the same in all groups (Table 2).

**Table 2:** Operative characteristics and wound complications.

Parameters	MIn = 406	TIn = 137	MMIn = 153	*P* value
Sutures				<.001
PDS 1 Plus	264 (65)	58 (42.3)	99 (64.7)	
PDS 2–0 Plus	142 (35)	79 (57.7)	54 (35.3)	
Drainage	393 (96.8)	135 (98.5)	148 (96.7)	.55
Total wound complications [n (%)]^∗^	136 (33.5)	31 (22.6)	50 (32.7)	.04
*Incisional hernia*	34 (8.4)	15 (10.9)	16 (10.5)	.97
*Fascial dehiscence*	33 (8.1)	5 (3.6)	12 (7.8)	.58
*SSI*	112 (27.6)	23 (16.8)	39 (25.5)	.04
Wound complications after re-incision [n (%)]^∗^				
*Incisional hernia*	16 (7.8)	9 (14.7)	12 (12)	.58
*Fascial dehiscence*	17 (8.3)	3 (4.9)	7 (7)	.2
*SSI*	55 (26.8)	7 (11.4)	21 (21)	.04
Wound intervention	102 (25.1)	23 (16.8)	41 (26.8)	.08
Reoperation due to wound infection	55 (13.5)	11 (8)	20 (13.1)	.23
Number of Reoperations				.3
≤3	45 (11.1)	8 (5.8)	18 (11.8)	
>3	12 (3)	3 (2.2)	2 (1.3)	

### 3.3 Comparing incidence of fascial dehiscence and incisional hernia between primary and re-incisions

**Figure 1:**
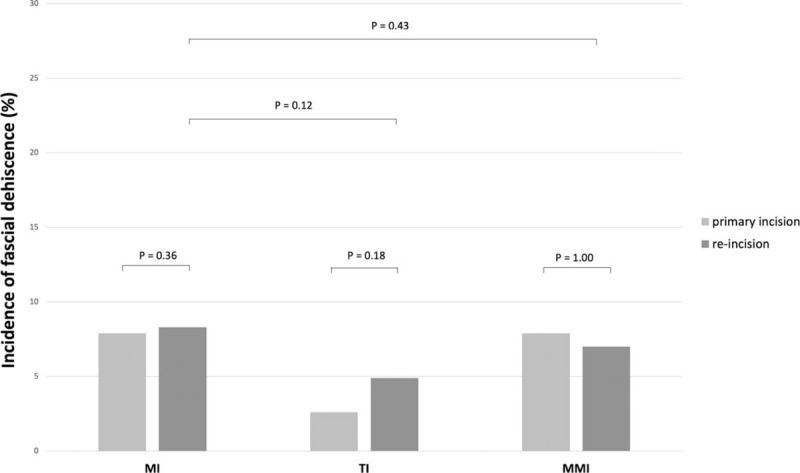
Incidence of fascial dehiscence after primary incision and re-incision of MI, TI, and MMI. MI = midline incision, MMI = modified Makuuchi incision, TI = transverse incision.

Overall, the incidence of fascial dehiscence was 7.6% (n = 53). After MI, in 33 cases a fascial dehiscence occurred (8.1%), however, there was no significantly difference (*P* = .36) between a primary MI (n = 16, 8%) or re-incision (n = 17, 8.3%). The lowest rate of fascial dehiscence (n = 5, 3.6%) occurred in TI group, the rate of fascial dehiscence after MMI was 10.5% (n = 16). In both groups, no difference between primary incision and re-incision could be detected (TI: *P* = 0.18; MMI: *P* = 1.00). The data are shown in Figure 1.

**Figure 2:**
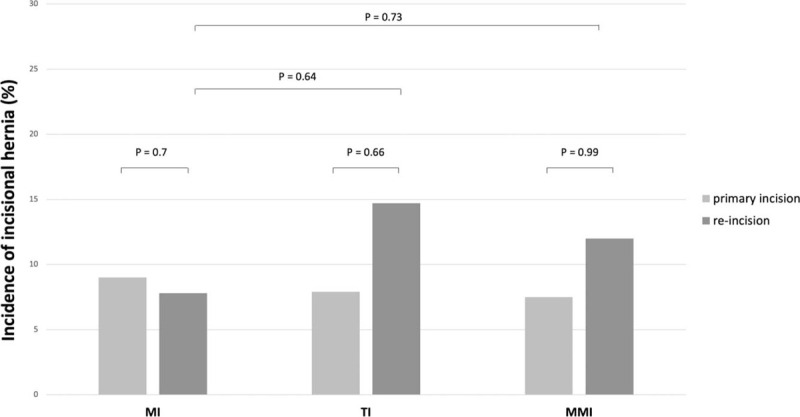
Incidence of incisional hernia after primary incision and re-incision of MI, TI, and MMI. MI = midline incision, MMI = modified Makuuchi incision, TI = transverse incision.

The overall incidence of incisional hernias was around 10%. After TI and MMI, the rate of incisional hernias slightly increased in cases of re-incision, however the differences were not statistically significant (TI: *P* = 0.66; MMI: *P* = .99). The incidence of incisional hernias after primary MI and re-incision were quite similar (8.9% vs 7.8%, *P* = .7). Comparing re-incisions via MI and TI or MI and MMI, the rate of incisional hernias was not significantly different (*P* = .12 and *P* = .43). All data are shown in Figure 2.

**Figure 3:**
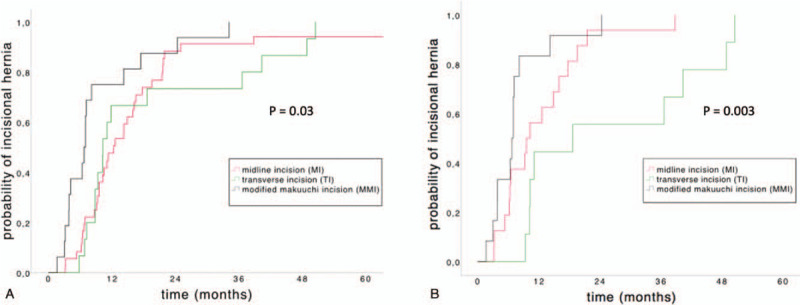
Kaplan–Meier curves of the probability of incisional hernias (“time to hernia” [TTH]). (A) TTH after MI (median TTH = 355 days, IQR 264–523 days), TI (median TTH 309 days, IQR 265–831 days) and MMI (median TTH = 206 days, IQR 117–290). **(B)** TTH after re-incision via MI (median TTH = 298 days, IQR 189–493 days), TI (median TTH = 561, IQR 309–1212 days) and MMI (median TTH = 206, IQR 117–290 days). IQR = interquartile range, MI = midline incision, MMI = modified Makuuchi incision, TI = transverse incision, TTH = time to hernia.

The interval from index operation to occurrence of incisional hernia is significantly shorter after MMI (median 206 days, IQR 117–290 days, *P* = .03) than after MI (median 355 days, IQR 264–523 days) or TI (median 309 days, IQR 265–831 days). In cases of re-incision, the TTH is also significantly shorter after MMI (median 206 days, IQR 117–290 days, *P* = .003) than after MI (median 298 days, IQR 189–493 days) or TI (median 561 days, IQR 309–1212 days). The Kaplan-Meier curves for probability of incisional hernia are shown in Figure 3.

### 3.4 Univariate and multivariate analysis of risk factors for developing wound complications

The uni- and multivariate analysis considered patient-related and operation-related risk factors for all cases and each type of incision separately.

In the MI subgroup (Table 3), BMI ≥25 kg/m^2^ is a risk factor for SSI in both univariate analysis (*P* = .02) and multivariate analysis (odds ratio [OR] 1.685, confidence interval [CI] 1.030–2.699, *P* = .04). For all others patient- or operation-related risk factors could be detected no significant influence neither in univariate analysis nor in multivariate analysis.

**Table 3:** Uni- and multivariate analysis of risk factors for wound complications after median incision (MI).

	Univariate analysis	Multivariate analysis		
Parameters	*P* value	relative OR	95% CI	*P* value
Incisional hernia				
BMI ≥25kg/m^2^	.04	2.019	0.897–4.545	.42
Diabetes	.43	0.598	0.237–1.511	.28
Nicotine abuse	.8	0.993	0.415–2.375	.99
Alcohol abuse	.39	0.621	0.239–1.612	.33
Previous laparotomy	.68	0.537	0.203–1.422	.21
Previous laparotomy via same incision	.7	1.408	0.477–4.156	.54
PDS 2–0	.48	0.664	0.294–1.500	.33
Drainage	1.0	n/a	n/a	1.0
Fascial dehiscence				
BMI ≥25kg/m^2^	.3	1.537	0.736–3.210	.25
Diabetes	.48	0.873	0.355–2.408	.87
Nicotine abuse	.58	0.694	0.483–2.985	.69
Alcohol abuse	.1	0.1	0.870–4.918	.1
Previous laparotomy	.9	0.996	0.356–2.798	1.0
Previous laparotomy via same incision	.36	0.298	0.595–5.719	.29
PDS 2–0	.35	0.105	0.863–4.755	.11
Drainage	1.0	n/a	n/a	1.0
SSI				
BMI ≥25kg/m^2^	.02	1.658	1.030–2.699	.04
Diabetes	.59	1.308	0.767 – 2.232	.32
Nicotine abuse	.38	1.227	0.698–2.160	.48
Alcohol abuse	.12	1.497	0.864–2.593	.15
Previous laparotomy	.73	0.732	0.395–1.357	.32
Previous laparotomy via same incision	.12	1.931	0.982–3.797	.06
PDS 2–0	.1	0.807	0.476–1.370	.43
Drainage	.38	0.700	0.196–2.504	.58

In the TI subgroup (Table 4), no significant influence of patient- or operation-related risk factors in uni- and multivariate analysis was detected for occurrence of incisional hernia and fascial dehiscence. In the univariate analysis of risk factors for developing SSI's, a previous TI was a significant risk factor (*P* = .04), however which could not be confirmed in multivariate analysis (OR 2.264, CI 0.562–9.119, *P* = .25).

**Table 4:** Uni- and multivariate analysis of risk factors for wound complications after transverse incision (TI).

	Univariate analysis	Multivariate analysis		
Parameters	*P* value	relative OR	95% CI	*P* value
Incisional hernia				
BMI ≥25kg/m^2^	.13	3.985	0.875–18.146	.07
Diabetes	.97	0.950	0.294–3.071	.93
Nicotine abuse	.14	0.190	0.022–1.640	.13
Alcohol abuse	.78	2.009	0.449–8.996	.36
Previous laparotomy	.21	1.900	0.549–6.579	.31
Previous laparotomy via same incision	.66	1.451	0.263–8.004	.67
PDS 2–0	.46	2.613	0.645–10.579	.18
Drainage	1.0	n/a	n/a	1.0
Fascial dehiscence				
BMI ≥25kg/m^2^	.53	3.566	0.365–34.806	.27
Diabetes	.54	0.950	0.050–4.610	.52
Nicotine abuse	1.0	n/a	n/a	1.0
Alcohol abuse	1.0	n/a	n/a	1.0
Previous laparotomy	.49	1.900	0.067–9.327	.85
Previous laparotomy via same incision	.16	1.451	0.280–55.23	.31
PDS 2–0	.43	2.613	0.078–5.039	.66
Drainage	1.0	n/a	n/a	1.0
SSI				
BMI ≥25kg/m^2^	.19	2.348	0.860–6.412	.1
Diabetes	.47	1.080	0.403 – 2.891	.88
Nicotine abuse	.84	0.917	0.270 – 3.112	.89
Alcohol abuse	.61	0.556	0.136–2.273	.41
Previous laparotomy	.21	1.201	0.384–3.759	.75
Previous laparotomy via same incision	.04	2.264	0.562–9.119	.25
PDS 2–0	.3	0.813	0.274–2.409	.71
Drainage	.25	0.381	0.019–7.722	.53

In analysis of influencing variables for occurrence of the different wound complications after MMI (Table 5), placing an intra-abdominal drainage could be detected as a protective factor for reducing fascial dehiscence (OR 0.115, CI 0.015–0.855, *P* = .04). In addition, no other patient- or operation-related significant risk factors were detected in univariate or multivariate analysis.

**Table 5:** Uni- and multivariate analysis of risk factors of wound complications after modified Makuuchi incision (MMI).

	Univariate analysis	Multivariate analysis		
Parameters	*P* value	relative OR	95% CI	*P* value
Incisional hernia				
BMI ≥25kg/m^2^	.08	4.409	0.877–22.1	.07
Diabetes	.33	0.570	0.118–2.764	.49
Nicotine abuse	.38	0.411	0.049–3.452	.41
Alcohol abuse	.18	0.266	0.033–2.146	.21
Previous laparotomy	.4	1.556	0.418–5.790	.51
Previous laparotomy via same incision	.99	1.085	0.259–4.539	.91
PDS 2–0	.166	2.186	0.681–7.014	.19
Drainage	1.0	n/a	n/a	1.0
Fascial dehiscence				
BMI ≥25kg/m^2^	.45	1.314	0.357–4.841	.68
Diabetes	.1	0.570	0.400 – 7.859	.45
Nicotine abuse	1.0	n/a	n/a	1.0
Alcohol abuse	.43	0.266	0.048–3.760	.44
Previous laparotomy	.6	1.556	0.210–4.194	.93
Previous laparotomy via same incision	.99	0.791	0.074–2.835	.4
PDS 2–0	.08	2.186	0.019–1.375	.1
Drainage	.02	0.115	0.015–0.855	.04
SSI				
BMI ≥25kg/m^2^	.3	2.079	0.840–5.150	.11
Diabetes	.14	1.452	0.561–3.755	.44
Nicotine abuse	.38	2.002	0.679–5.933	.21
Alcohol abuse	.22	1.428	0.546–3.735	.47
Previous laparotomy	.08	0.773	0.321–1.861	.57
Previous laparotomy via same incision	.12	0.345	0.106–1.125	.08
PDS 2–0	.69	0.624	0.263–1.480	.29
Drainage	.46	0.317	0.046–2.172	.24

## 4 Discussion

There is ongoing debate as to which suturing techniques and suture materials are best for achieving optimal abdominal wound closure while minimizing postoperative wound complications. A midline laparotomy is the most common incision used in visceral surgery. In general, incisional hernias or wound infections after laparotomy represent serious postoperative problems. After MI, incisional hernias occur in 10% to 15% of cases and wound infections in 20% to 30%.^[1,9]^ In this study, the rate of incisional hernias was slightly lower at 8.4%. The rate of local wound infections was similar with about 30%. However, in previous randomized-controlled studies with adequate follow-up time, the rates of wound associated complications were significantly higher. In a 3-year follow-up of 2 randomized controlled studies, it was demonstrated that even after more than a year, the risk of incisional hernias still increases significantly.^[10]^ So it is quite possible, that the rate of incisional hernias after MI in this study could be higher, if the follow up time would be longer than the median follow-up time of 600 days. The incidence of fascial dehiscence is reported up to 5% after elective midline laparotomy.^[11,12]^ However, our results were slightly higher (8.1%) which can be explained by mostly performed clean-contaminated operations and its associated higher incidence of epifascial SSI.

A re-incision via MI seems not to increase the risk for wound complications, especially the stability of the abdominal wall closure does not seem to be influenced by a midline relaparotomy.

In comparing MI and TI, several studies have reported lower pain and better pulmonary recovery after TI.^[13,14]^ Grantcharov and Rosenberg reported a lower morbidity in the early postoperative period and a lower incidence of late incisional hernias after TI compared with MI.^[15]^ In the POVATI trial on the other hand, more wound infections were reported after TI than after MI.^[7]^ In our study, the rate of SSI (27.6% vs 16.8%, *P* = .04) after TI were significantly lower than for MI. We often use MI for open colorectal surgery and TI for upper abdominal surgery. The increased rate of SSI after MI compared to TI can probably be attributed to bacterial contamination within colorectal surgery. Despite the fact that both the fascia and rectus muscle are completely severed again in a repeated TI, this does not seem to be an additional risk factor for the development of fascial dehiscence or incisional hernia even if different suture material is used for abdominal wall closure in the case of relaparotomy. Although in this study a marginal increased rate of incisional hernia after transverse re-incision was seen, but a previous laparotomy represented no risk factor developing incisional hernia in uni- and multivariate analysis. For pancreatic resections in particular, which are usually performed by TI, the placement of an intra-abdominal drain has been discussed.^[16]^ At least in the uni- and multivariate analysis, a perioperative intra-abdominal drain does not seem to be a risk factor for disturbed wound healing, or for the occurrence of fascial dehiscence or incisional hernia.

Data for wound complications after MMI are limited. In our study, the rate of incisional hernias (10.5%) was similar to that reported by Chang et al (10.9%)^[17]^ with comparable postoperative follow-up time. In contrast, the incidence of hernias in the study by Togo et al was only 5.4%.^[18]^ In our study, intraoperative drain placement during MMI was a significant factor in preventing fascial dehiscence (Table 5).

MMI is often used for major liver resection, which may be associated with larger amounts of ascites and increased intra-abdominal pressure. Therefore, inserting a drain intraoperatively after MMI always seems to be useful. In cases of living donor liver transplantation, Shu et al reported lower rates of wound infections or seroma after MMI^[8]^ than in this study. While Chang et al. also reported relatively low rates of wound seroma, they used looped suture for closing the abdominal wall.^[17]^ According to the results of uni- and multivariate analysis, a repeated MMI or MMI after previous laparotomy are no risk factors developing fascial dehiscence or incisional hernia, although the incidence of incisional hernias after re-incision via MMI is slightly higher (7.5% vs 12%). It is of clinical importance that the abdominal wall stability would not be affected by a previous laparotomy. Interestingly, the disease-free survival regarding the occurrence of incisional hernias as well after primary MMI (*P* = .03) as after re-incision via MMI (*P* = .003) is significantly shorter than for TI or MI.

Although, it was reported, that relaparotomies carry a higher risk for long-term complications such as incisional hernia,^[19]^ our data demonstrate other findings. Neither for re-incision via MI, TI or MMI, a significantly increased risk of fascial dehiscence or incisional hernia could be demonstrated. Maybe, current prospective trails can provide more evidence in this topic.^[20,21]^

The limitations of this study are its retrospective character and the heterogeneity within the study groups. In particular, allocation of the operative procedures performed to the 3 different incisions and comparing incisions for clean or clean-contaminated operations weakens the statistical results. Nevertheless, our data are comparable with previous prospective studies and provide additional evidence especially with respect to relaparotomies and its wound complication rate. Furthermore, the heterogeneity in the use of suture material does not allow any conclusion regarding wound complications.

## 5 Conclusion

This study demonstrates, that the incidence of fascial dehiscence and incisional hernia after relaparotomy is not relevantly increased. The stability of the abdominal wall is apparently not affected by repeated MIs, TIs, and MMIs, even by relaparotomy via the same incision. Therefore, the type of laparotomy, especially a relaparotomy, can be chosen based on the surgeon's preference and the planned surgical procedure without worrying about increased wound complications.

## Author contributions

**Conceptualization:** Sebastian Hempel and Marius Distler.

**Data curation:** Sebastian Hempel, Anne Kalauch, Thilo Welsch, Jürgen Weitz, Marius Distler.

**Formal analysis:** Florian Oehme, Steffen Wolk.

**Supervision:** Marius Distler.

**Visualization:** Sebastian Hempel.

**Writing – original draft:** Sebastian Hempel.

**Writing – review & editing:** Thilo Welsch, Jürgen Weitz, Marius Distler.
